# Comparison of two echocardiography-based methods for evaluating pediatric left ventricular diastolic dysfunction

**DOI:** 10.3389/fped.2023.1206314

**Published:** 2023-09-04

**Authors:** Xue Xiang, Xu Zhu, Min Zheng, Yi Tang

**Affiliations:** Department of Ultrasound, Children’s Hospital of Chongqing Medical University, National Clinical Research Center for Child Health and Disorders, Ministry of Education Key Laboratory of Child Development and Disorders, Chongqing Key Laboratory of Pediatrics, Chongqing, China

**Keywords:** children, heart failure, left ventricular diastolic function, left ventricular diastolic dysfunction, *Z* value

## Abstract

**Objectives:**

To investigate the consistency between the 2016 America Society of Echocardiography (ASE)/European Association of Cardiovascular Imaging (EACVI) guideline-based recommendations and the body surface area (BSA)-transformed *Z* value-based cut-off for the assessment of left ventricular diastolic function (LVDF) in children.

**Methods:**

Clinical data of children with heart failure (HF) and those with a high risk of HF and a low risk of HF were collected from the Children's Hospital of Chongqing Medical University between March 2021 and October 2022. The mitral annular *e*′ velocity, lateral *E*/*e*′ ratio, left atrial volume index, and peak tricuspid regurgitation velocity were detected by Echocardiography. The cut-off values recommended by the 2016 ASE/EACVI guidelines and the cut-off value based on the BSA-transformed *Z* value were used to evaluate LVDF. The consistencies and differences of the two criteria were compared.

**Results:**

A total of 132 children with HF, 189 with a high risk of HF, and 231 with a low risk of HF, were enrolled. The consistency of the two criteria in evaluating LVDF in children with HF and with high risk of HF was moderate, with weighted kappa coefficients of 0.566 and 0.468, respectively (*P* < 0.001). The positivity rate of left ventricular diastolic dysfunction (LVDD) with *Z* value-based criteria (HF group, 23.5%; high-risk group, 8.5%) was higher than that with guideline-based criteria (HF group, 15.6%; high-risk group, 3.2%). In children with a low risk of HF, no case with LVDD was found. The consistency between the two criteria for grading the degree of LVDD was moderate, with a kappa coefficient of 0.522 (*P* = 0.001). The degree of LVDD according to the *Z* value-based criteria was higher than that of the guideline-based criteria (*P* = 0.004).

**Conclusions:**

The *Z* value-based criteria used to evaluate LVDD in children with HF and high risk of HF may be more conducive to the early identification of LVDD, thereby permitting the possibility of early treatment intervention.

## Introduction

Heart failure (HF) is a major threat to global public health, affecting approximately 43 million people worldwide (1). Many clinical epidemiological investigations have shown that over half of HF is heart failure with preserved ejection fraction (diastolic HF) (1, 2). Some studies also show that more than 90% of patients with HF have diastolic dysfunction unrelated to left ventricular ejection fraction (LVEF) (3). Growth retardation or cachexia in children with diastolic HF is more severe than in those with systolic HF (4). Because the treatment methods for systolic and diastolic HF differ, early identification of diastolic dysfunction in patients with HF is beneficial for prompt clinical intervention and prognosis improvement. In 2016, the American Society of Echocardiography (ASE) and the European Association of Cardiovascular Imaging (EACVI) jointly published the “Recommendations for Evaluation of Left Ventricular Diastolic Function by Echocardiography” (5). A simple and feasible procedure for evaluating left ventricular diastolic dysfunction (LVDD) using Echocardiography was proposed, and the cut-off value of each index was determined. However, the cut-off value recommended in that guideline is mainly based on adults and has not been verified in children. These guidelines emphasise that this method is not necessarily applicable to children. If there is an appropriate age-dependent cut-off, then use of that cut-off can be considered in the evaluation of LVDD.

The size of the heart is affected not only by disease but also by many confounding factors, including body size, age, genetics, sex, race, growth and developmental patterns, among which height and weight have been the most influential determinants of the size of the heart in children, and body surface area (BSA) is considered to reflect the growth and development of children more than height and weight alone (6). The normal reference value range of children's hearts changes with age, which in turn presents communication challenges when conveying this intricate description among researchers. In 2010, the Pediatric Measurement Writing Group of the Pediatric and Congenital Heart Disease Committee of the American Society of Echocardiography recommended that the *Z* value be selected to distinguish the normal and abnormal values of children's hearts in the ‘Quantitative Analysis Guide for Pediatric Echocardiography’. When the *Z* value is >2 or <−2, the difference between the measured value of the patient and the average value of the reference population is more than twice the standard deviation of the average value, which exceeds the normal range of 95%, and is frequently characterised as clinically “abnormal” (7).

Therefore, literature on how consistent the standardised BSA *Z*-value is, the cut-off value recommended by the guidelines for the diagnosing of LVDD in children, and which cut-off value is more suitable for children is scarce. Consequently, we aimed to assess the consistency and differences between the cut-off value recommended by the 2016 ASE/EACVI guidelines and the cut-off value based on the BSA-standardised *Z* value used in the diagnosis of LVDD in children with HF and those with high risk or low risk of HF, and the consistency and difference between the two standards were compared. The results showed that in children with HF and in those with a high risk of HF, the positive rate for the diagnosis of LVDD by using the cut-off value based on the BSA-standardized *Z* value is higher than the cut-off value based on the guidelines, and there is no significant difference between the two cut-off value standards in the diagnosis of LVDD in children with a low risk of HF, suggesting that using the cut-off value based on the BSA-standardized *Z* value may be more conducive to the early diagnosis of LVDD in children with HF and those with a high risk of HF.

## Research method

### Research object

Children with HF and high risk or low risk of HF who were treated in the Children's Hospital of Chongqing Medical University between March 2021 and October 2022 were studied. The diagnostic criteria for HF are described in the “Recommendations for the Diagnosis and Treatment of HF in Children (2020 revised edition)” (7). The exclusion criteria included mitral valve disease, right ventricular outflow tract obstruction and pulmonary artery stenosis. The inclusion criteria for children with a high risk of HF are the following basic diseases or medical history: congenital heart disease, acquired heart disease, cardiomyopathy, myocarditis, coronary artery disease, arrhythmia, toxic drug injury, hypertension, diabetes, obesity, infection, malnutrition, severe anaemia, kidney disease, cardiovascular disease-related family history and previous history (8–11). Children with low risk of HF were referred to those who did not have the aforementioned basic diseases or medical history. This study was approved by the Ethics Committee of Children's Hospital of Chongqing Medical University (96/2021).

### Data collection

**Figure 1:**
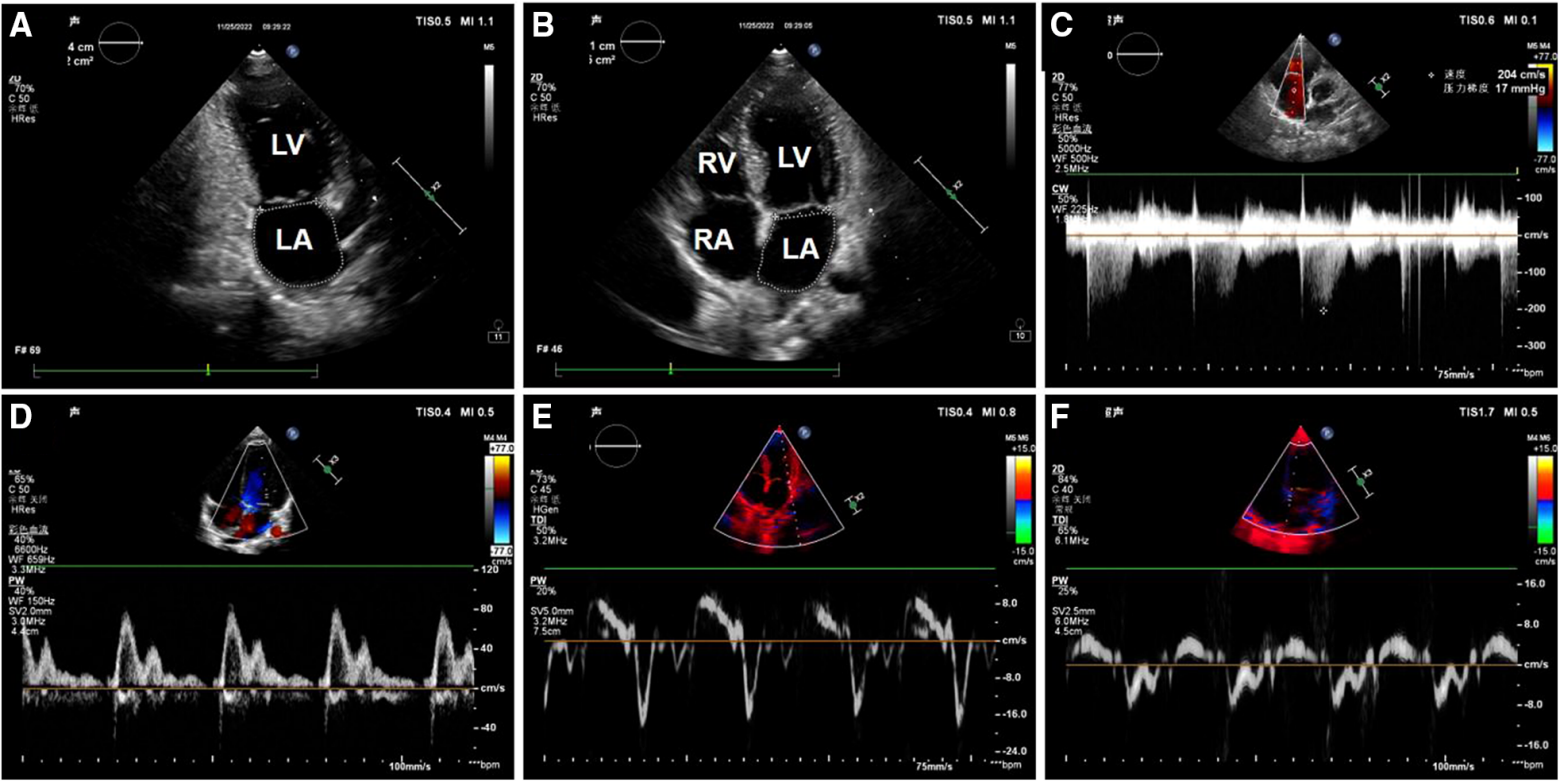
Image of left ventricular diastolic function measurement in one of the patients. (A) Apical two-chamber cardiac view: The area and long diameter of left atrial were measured; (**B**) apical four-chamber cardiac view: The area and long diameter of left atrial were measured; (**C**) spectrum of tricuspid regurgitation; (**D**) mitral valve flow pattern; (**E**) the mitral annular lateral wall TDI; (**F**) the mitral annular septal TDI. LV, left ventricle; LA, left atrium; RV, right ventricle; RA, right atrium.

Clinical data was collected including age, sex, height, weight, Ross score, cause of HF, basic disease and BNP levels, were collected. The Ross scoring standards are described in the 2013 Canadian guidelines (12). Echocardiographic data were collected by a PHILIPS EPIQ 7C or CX50 ultrasonic diagnostic instrument and S5-1 and S8-3 phased array probes, with frequencies of 1–5 MHz and 3–8 MHz, respectively. The main collected data included the following measurements (Figure 1): continuous wave Doppler measurement of the peak velocity of tricuspid regurgitation; pulse-wave Doppler measurement of the peak velocity of early diastolic mitral valve (*E*), and the peak velocity of late diastolic mitral valve (*A*); the mitral annular septal *e*′, mitral annular septal *a*′, mitral annular lateral wall *e*′, and mitral annular lateral wall *a*′, which were measured by tissue Doppler; measurement of left atrial maximum volume index by biplane area-length method (13); and the left ventricular end-diastolic diameter and end-systolic diameter were measured by M-mode Echocardiography. The left ventricular end-diastolic volume index (LVEDVI) and left ventricular ejection fraction (LVEF) were calculated using the Teichholz formula.

### Determination of the cut-off value of the left ventricular diastolic function index by Echocardiography

According to the normal reference value of Echocardiography based on BSA, when the *Z* value is >2 or <−2, as described in the paediatric ultrasound diagnosis edited by Guoying Huang and Bei Xia (14), it means that the measurement has exceeded the cut-off value and is characterised as “abnormal”. The critical BSA values are listed in Supplementary Table S1. Because there is no normal reference value for the peak velocity of tricuspid regurgitation is 2.8 m/s according to the 2016 guidelines. The indices for evaluating LVDF and the corresponding cut-off values based on the guidelines were mitral annular septal wall *e*′ < 7 cm/s, mitral annular lateral wall *e*′ < 10 cm/s, mitral annular septal *e*′ > 15, left atrial maximum volume index >34 mL/m^2^, and tricuspid regurgitation peak velocity >2.8 m/s. Diagnosis of LVDD was as follows: ≤1 abnormal indicators can be defined as normal LVDF, 2 abnormal indicators was defined as uncertain LVDF, and ≥3 abnormal indicators can be defined as LVDD. For children with HF with reduced LVEF, the grading criteria for LVDD are described in the 2016 ASE/EACVI guidelines (5).

### Statistical analysis

Classification variables were expressed as frequencies and percentages, and the chi-square test or Fisher's exact probability method was used for intergroup comparisons. Continuous variables with a normal distribution were expressed as mean ± standard deviation. Intergroup comparisons were performed by analysis of variance, and multiple comparisons were corrected by the Bonferroni method. Continuous variables with skewed distributions were expressed as median and interquartile interval, and intergroup comparisons were performed by the rank sum test. The consistency of the diagnosis results were expressed by the kappa coefficient (two levels) or the weighted kappa coefficient (multiple levels), and the difference test was performed by paired chi-square tests (two levels) or paired rank sum tests (multiple levels). A kappa coefficient of <0.4 was considered to indicated poor consistency, while a kappa coefficient of between ≥0.4 and <0.7 was considered to indicate moderate consistency and ≥0.7 was considered to indicated good consistency; a *P* value of <0.05 was considered statistically significant.

## Results

### Basic clinical characteristics in each group

A total of 132 children with HF, 189 children with a high risk HF, 231 children with a low risk of HF, 67 patients with grade II heart function (50.8%), 48 patients with grade III heart function (36.4%), and 17 patients with grade IV heart function (12.9%) were included in this study. The basic clinical characteristics of each group were analysed (Table 1). The results of the basic clinical characteristics of each group were as follows:

**Table 1:** Basic clinical characteristics of children in each group.

		HF group (*n* = 132)	High risk of HF (*n* = 189)	Low risk of HF (*n* = 231)	*P*-value
Age [median (IQR)], years		0.63 [0.36, 4.56]	3.58 [2.00, 6.50]	3.00 [1.00, 5.96]	<0.001▴
Sex (*n*, %)	Male	65 (49.2)	92 (48.7)	116 (50.2)	0.95△
	Female	67 (50.8)	97 (51.3)	115 (49.8)	
BSA [median (IQR)], m^2^		0.35 [0.27, 0.73]	0.64 [0.49, 0.84]	0.59 [0.42, 0.81]	<0.001▴
ROSS grading (*n*, %)	II	67 (50.8)	/	/	/
	III	48 (36.4)	/	/	/
	IV	17 (12.9)	/	/	/
BNP [median (IQR)], pg/ml		191 [64, 654]	/	/	
Basic disease (*n*, %)	Congenital heart disease	83 (62.9)	154 (81.5)	/	/
	Cardiomyopathy	16 (12.1)	2 (1.1)	/	/
	Arrhythmia	11 (8.3)	1 (0.5)	/	/
	Myocarditis	6 (4.5)	1 (0.5)	/	/
	Primary pulmonary hypertension	4 (3.0)	0 (0.0)	/	/
	Coronary artery disease	3 (2.3)	2 (1.1)	/	/
	Pneumonia	2 (1.5)	9 (4.8)	/	/
	Sepsis	2 (1.5)	4 (2.1)	/	/
	Renal insufficiency	1 (0.8)	11 (5.8)	/	/
	Chemotherapy	1 (0.8)	2 (1.1)	/	/
	Other	3 (2.3)	3 (1.6)	/	/
LVEDVI [median (IQR)], ml/m^2^		86 [60, 121]	67 [54, 82]	61 [57, 67]	<0.001▴
LVEF [median (IQR)], %		61 [51, 68]	66[62, 69]	66 [63, 69]	<0.001▴
Maximum left atrial volume index [median (IQR)], ml/m^2^		23 [16, 34]	20[16, 26]	16 [14, 20]	<0.001▴
TRV [median (IQR)], m/s		2.51 [2.08, 3.09]	2.14 [2.01, 2.40]	2.05 [2.01, 2.16]	<0.001▴
Mitral annular septal *e*′ [median (IQR)], m/s		0.08 [0.06, 0.10]	0.10 [0.09, 0.12]	0.12 [0.10, 0.14]	<0.001▴
Lateral wall of mitral annulus *e*′ [median (IQR)], m/s		0.10 [0.07, 0.12]	0.12 [0.09, 0.14]	0.14 [0.11, 0.15]	<0.001▴
Mitral annular septal *E*/*e*′ [median (IQR)]		12 [9, 16]	9 [8, 12]	9[8, 10]	<0.001▴

BSA, body surface area; BNP, B-type brain natriuretic peptide; HF, heart failure; LVEDVI, left ventricular end-diastolic volume index; LVEF, left ventricular ejection fraction; TRV, peak velocity of tricuspid regurgitation; ▴, Rank sum test; △, chi-square tests.

LVEDVI, maximum volume index of the left atrium, peak velocity of tricuspid regurgitation, and mitral annular interval *E*/*e*′ showed a downwards trend in the HF, high-risk HF and low-risk HF groups: Particularly, LVEDVI values for HF, high-risk, and low-risk HF groups were 80 [60, 121], 67 [54, 82], and 61 [57, 67] mL/m^2^, respectively. The maximum volume index of the left atrium was 23 [16, 34], 20 [16, 26], and 16 [14, 20] mL/m^2^, respectively. The peak velocity of tricuspid regurgitation values for HF, high-risk, and low-risk HF groups were 2.51 [2.08, 3.09], 2.14 [0.01, 2.40], and 2.05 [0.01, 2.16] m/s, respectively. Mitral annular septal *E*/*e*′ values for HF, high-risk, and low-risk HF groups were 12 [9, 16], 9 [8, 12], and 9 [8, 10], respectively.

Mitral annular septal *e*′ and mitral annular lateral wall *e*′ in the HF group and the high-risk and low-risk HF groups showed an upwards trend. Notably, mitral annular septal *e*′ values for HF, high-risk, and low-risk HF groups were 0.08 [0.06, 0.10], 0.1 [0.09, 0.12] m/s, and 0.12 [0.1, 0.14] m/s, respectively. The lateral wall *e*′ of the mitral valve ring values for HF, high-risk, and low-risk HF groups were 0.1 [0.07, 0.12], 0.12 [0.09, 0.14] m/s, and 0.14 [0.11, 0.15] m/s, respectively.

### Comparison of the consistency and difference between the two standards in assessing the abnormality of a single index of LVDF

Among the 132 children with HF (Table 2), 94 children were consistent between the two standards in the evaluation of the left atrial maximum volume index, 38 children were inconsistent; 100 children were consistent between the two standards in the evaluation of the mitral annular septal or lateral wall *e*′, and 32 children were inconsistent; 109 children were consistent between the two standards in the evaluation of mitral annular septal *E*/*e*′, while 23 children were inconsistent. Based on the guidelines, 33 cases (25.0%) were identified as having abnormal left atrial maximum volume index, 67 cases (50.8%) were identified as having abnormal mitral annular septal or lateral wall *e*′ abnormalities, and 40 cases (30.3%) were identified as having abnormal mitral annular septal *E*/*e*′. Based on the *Z* value criteria, 71 cases (53.8%) were identified as having abnormal left atrial maximum volume index, 59 cases (44.7%) were identified as having abnormal mitral annular septal or lateral wall *e*′, and 41 cases (31.1%) were identified as having abnormal mitral annular septal *E*/*e*′. In the HF group, the kappa coefficient of the two criteria that identified the abnormality of left atrial maximum volume index was 0.445 (*P* < 0.001), the mitral annular septal or lateral wall *e*′ was 0.516 (*P* < 0.001), and the mitral annular septal *E*/*e*′ was 0.59 (*P* < 0.001). The consistency strength was medium. The positive rate of identifying the abnormality of the left atrial maximum volume index based on the *Z*-value was higher than that of the guideline standard (*P* < 0.001), while the positive rate of the abnormality of mitral septal or lateral wall *e*′ and mitral septal *E*/*e*′ was not significantly different between the two criteria.

**Table 2:** Comparison of single index abnormalities of LVDF evaluated by two standards.

		Criteria based on *Z* value	Criteria based on Guideline			Kappa value	Kappa test *P*-value	Paired chi-square test *P*-value
			Normal	Abnormal	Total			
HF	Maximum LAVI (*n*, %)	Normal	61 (46.2)	0 (0.0)	61 (46.2)	0.445	<0.001	<0.001
		Abnormal	38 (28.8)	33 (25.0)	71 (53.8)			
		Total	99 (75.0)	33 (25.0)	132 (100)			
	Septal or lateral *e*′ (*n*, %)	Normal	53 (40.2)	20 (15.2)	73 (55.3)	0.516	<0.001	0.215
		Abnormal	12 (9.1)	47 (35.6)	59 (44.7)			
		Total	65 (49.2)	67 (50.8)	132 (100)			
	Septal *E*/*e*′ (*n*, %)	Normal	80 (60.6)	11 (8.3)	91 (68.9)	0.59	<0.001	1
		Abnormal	12 (9.1)	29 (22.0)	41 (31.1)			
		Total	92 (69.7)	40 (30.3)	132 (100)			
High-risk of HF	Maximum LAVI (*n*, %)	Normal	90 (47.6)	0 (0.0)	90 (47.6)	0.194	<0.001	<0.001
		Abnormal	79 (41.8)	20 (10.6)	99 (52.4)			
		Total	169 (89.4%)	20 (10.6)	189 (100)			
	Septal or lateral *e*′ (*n*, %)	Normal	113 (59.8)	23 (12.2)	136 (72.0)	0.462	<0.001	0.644
		Abnormal	19 (10.1)	34 (18.0)	53 (28.0)			
		Total	132 (69.8)	57 (30.2)	189 (100)			
	Septal *E*/*e*′ (*n*, %)	Normal	166 (87.8)	2 (1.1%)	168 (88.9)	0.571	<0.001	0.022
		Abnormal	11 (5.8)	10 (5.3)	21 (11.1)			
		Total	177 (93.7)	12 (6.3)	189 (100)			
Low-risk of HF	Maximum LAVI (*n*, %)	Normal	170 (73.6)	0 (0)	170 (73.6)	0.048	0.018	<0.001
		Abnormal	59 (25.5)	2 (0.9)	61 (26.4)			
		Total	229 (99.1)	2 (0.9)	231 (100)			
	Septal or lateral wall *e*′ (*n*, %)	Normal	190 (82.3)	30 (13.0)	220 (95.2)	0.273	<0.001	<0.001
		Abnormal	3 (1.3)	8 (3.5)	11 (4.8)			
		Total	193 (83.5)	38 (16.5)	231 (100)			
	Septal *E*/*e*′	Normal	229 (99.1)	0 (0)	229 (99.1)	0.665	<0.001	1
		Abnormal	1(0.4)	1(0.4)	2(0.9)			
		Total	230(99.6)	1(0.4)	231(100)			

LAVI, left atrial volume index; LVDF, left ventricular diastolic function; HF, heart failure.

Among 189 children with high risk of HF (Table 2), 110 children were consistent between the two standards in the evaluation of the abnormal left atrial maximum volume index, and 79 children were inconsistent. The assessment of the mitral annular septal or lateral wall *e*′ was consistent in 147 patients, and the assessment of 42 patients was inconsistent; 176 children were consistent between the two standards in the evaluation of abnormal mitral annular septal *E*/*e*′, while 13 children were inconsistent. Based on the guidelines, 20 cases (10.6%) were identified as having abnormal left atrial maximum volume index, 57 cases (30.2%) were identified as having abnormal mitral annular septal or lateral wall *e*′, and 12 cases (6.3%) were identified as having abnormal mitral annular septal *E*/*e*′. Based on the *Z* value criteria, 99 cases (52.4%) were identified as having abnormal atrial maximum volume index, 53 cases (28.0%) were identified as having abnormal mitral annular septal or lateral wall *e*′ abnormalities, and 21 cases (11.1%) were identified as having abnormal mitral annular septal *E*/*e*′ abnormalities. In general, in the high-risk HF group, the kappa coefficient of the two criteria for identifying left atrial maximum volume index abnormalities was 0.194 (*P* < 0.001), and the consistency strength was poor; the mitral annular septal or lateral wall *e*′ was 0.468 (*P* < 0.001), and the consistency strength was medium; that for the mitral annulus interval *E*/*e*′ was 0.571 (*P* < 0.001), and the consistency strength was medium. Among them, the positive rates of identifying an abnormal left atrial maximum volume index or mitral annular septal *E*/*e*′ abnormalities based on the *Z* value criteria was higher than those of the guideline standard (*P* < 0.05), while the positive rate of identifying mitral annular septal or lateral wall *e*′ abnormalities based on the two standards was not significantly different.

Among 231 children with low risk HF (Table 2), 172 children were consistent between the two standards in the evaluation of the abnormal left atrial maximum volume index, while 59 children were inconsistent. The evaluation of the mitral annular septal or lateral wall *e*′ in 198 children was consistent, and 33 children were inconsistent. The evaluation of mitral annular septal *E*/*e*′ in 230 patients was consistent, and 1 child was inconsistent. Based on the guideline criteria, 2 patients (0.9%) were identified as having abnormal left atrial maximum volume index, 38 patients (16.5%) were identified as having abnormal mitral annular septal or lateral wall *e*′, and 1 patient (0.4%) was identified as having abnormal mitral annular septal *E*/*e*′. Based on the *Z* value criteria, 61 cases (26.4%) were identified as having abnormal left atrial maximum volume index, 11 cases (4.8%) were identified as having abnormal mitral annular septal or lateral wall *e*′ abnormalities, and 3 cases (0.9%) were identified as having abnormal mitral annular septal *E*′. In general, in the low-risk HF group, the kappa coefficient of the two criteria for identifying left atrial maximum volume index abnormalities was 0.048 (*P* < 0.001), and the consistency strength was poor, while that for the mitral annular septal or lateral wall *e*′ was 0.273 (*P* < 0.001), and the consistency strength was poor and that for mitral annulus interval *E*/*e*′ was 0.665 (*P* < 0.001), with moderate consistency strength. Among them, the positive rate of identifying the left atrium maximum volume index abnormalities based on *Z* value criteria was higher than that of the guidelines (*P* < 0.05).

### Comparison of the consistency and difference between the two standards in identifying LVDD in each group

Among the 132 children with HF (Table 3), 89 children were consistent between the two standards in the evaluation of LVDD, while 43 children were inconsistent. Among 189 children with a high risk of HF, 151 children were consistent between the two standards in the evaluation of LVDD, while 38 children were inconsistent. Among 231 children with low risk of HF, 227 children were consistent between the two standards in the evaluation of LVDD, while 4 children were inconsistent. In general, the weighted kappa coefficient for the evaluation of LVDD using the two standards was 0.566 in the HF group (*P* < 0.001), with moderate consistency strength. That in the high risk of HF group was 0.468 (*P* < 0.001), with moderate consistency strength, and that in low risk HF group was 0.326 (*P* < 0.001), with poor consistency strength.

**Table 3:** Comparison of two criteria in evaluating left ventricular diastolic dysfunction.

	Criteria based on *Z* value	Criteria based on Guideline				Kappa value	Kappa test *P*-value	Signed rank sum test *P*-value
		Normal LVDF	Uncertain LVDF	LVDD	Total			
HF (*n*, %)	Normal LVDF	55 (41.7)	8 (6.1)	1 (0.8)	64 (48.5)	0.566	<0.001	0.011
	Uncertain LVDF	15 (11.4)	18 (13.6)	4 (3.0)	37 (28.0)			
	LVDD	3 (2.3)	12 (9.1)	16 (12.1)	31 (23.5)			
	Total	73 (55.3)	38 (28.8)	21 (15.9)	132 (100)			
High risk of HF (*n*, %)	Normal	139 (73.5)	4 (2.1)	0 (0)	143 (75.7)	0.468	<0.001	<0.001
	Uncertain	18 (9.5)	9 (4.8)	3 (1.6)	30 (15.9)			
	Abnormal	5 (2.6)	8 (4.2)	3 (1.6)	16 (8.5)			
	Total	162 (85.7)	21 (11.1)	6 (3.2)	189 (100)			
Low risk of HF (*n*, %)	Normal	226 (97.8)	1 (0.4)	0	227 (98.3)	0.326	<0.001	0.317
	Uncertain	3 (1.3)	1 (0.4)	0	4 (1.7)			
	Total	229 (99.1)	2 (0.9)	0	231 (100)			

HF, heart failure; LVDF, left ventricular diastolic function; LVDD, left ventricular diastolic dysfunction.

Among 132 children with HF (Table 3), 73 (55.3%) were identified as having normal LVDF, 38 (28.8%) were identified as having uncertain LVDF, and 21 (15.6%) were identified as having LVDD according to the criteria of the guidelines. Based on the *Z* value criteria, 64 children (48.5%) were identified as having normal LVDF, 37 (28.0%) were identified as having uncertain LVDF, and 31 (23.5%) were identified as having LVDD. The overall difference was statistically significant (*P* = 0.011), suggesting that in the HF group, the positive rate of diagnosis of LVDD based on the *Z* value criteria was higher than that which was based on the guidelines.

Among 189 children with a high risk of HF (Table 3), 162 (85.7%) were identified as having normal LVDF, 21 (11.1%) were identified as having uncertain LVDF, and 6 (3.2%) were identified as having LVDD according to the criteria of the guidelines. Based on the *Z* value criteria, 143 cases (75.7%) were identified as having normal LVDF, 30 cases (15.9%) were identified as having uncertain LVDF, and 16 cases (8.5%) were identified as having LVDD. The overall difference was statistically significant (*P* < 0.001), suggesting that in the high-risk HF group, the positive rate of diagnosis of LVDD based on the *Z* value was higher than that based on the guidelines.

Among 231 children with a low risk of HF (Table 3), 229 children (99.1%) were identified as having normal LVDF, 2 (0.9%) had uncertain LVDF, and no cases of LVDD according to the criteria of the guidelines. Based on the *Z* value criteria, 227 children (98.3%) were identified as having normal LVDF, 4(1.7%) had uncertain LVDF, and none had LVDD. The overall difference was not statistically significant (*P* = 0.317), suggesting that in the low-risk HF group, there was no statistically significant difference between the two criteria in the diagnosis of LVDD.

### Comparison of the consistency and difference between the two standards in the classification of LVDD

According to the recommendations of ASE/EACVI in 2016, the LVDD of 44 children with HF and reduced LVEF (<55%) was graded, and the consistency of the two criteria for the classification of LVDD was analysed. The results (Table 4) showed that the classification of LVDD in 35 children was consistent between the two standards, whereas 9 children were inconsistent. The kappa coefficient of the two criteria for evaluating the LVDD grade was 0.522 (*P* = 0.001), with moderate consistency.

**Table 4:** Comparison of two criteria in the classification of LVDD.

Criteria based on *Z* value	Criteria based on guideline			Kappa value	Kappa test *P*-value	Paired chi-square test *P*-value
	LVDD grade I	LVDD grade II	Total			
LVDD grade I (*n*, %)	27 (61.4)	0 (0)	27 (61.4)	0.522	<0.001	0.004
LVDD grade II (*n*, %)	9 (20.5)	8 (18.2)	17 (38.6)			
Total	36 (81.8)	8 (18.2)	44 (100)			

LVDD, left ventricular diastolic dysfunction.

Among 44 children with HF with reduced LVEF, 36 (81.8%) children were identified as having LVDD grade I, and 8 (18.2%) were identified as having LVDD grade II based on the criteria of the guidelines; 27 (61.4%) children were identified as having LVDD grade I, and 17 (38.6%) were identified as having LVDD grade II based on the *Z* value. There was a statistically significant difference between the two criteria in assessing the grade of LVDD (*P* = 0.004), suggesting that the severity of LVDD based on the *Z* value criteria was higher than that based on the guidelines.

## Discussion

In this study, 132 children with HF, 189 children with a high risk of HF, and 231 children with a low risk of HF were evaluated for LVDF using two different criteria, namely, the cut-off value based on the BSA-transformed *Z* value and the cut-off value recommended by the guidelines (5). The consistency and positivity rate of LVDD between the two criteria were analysed. First, this study demonstrated that the consistency strength of the two criteria in diagnosing LVDD in children with HF and high risk of HF was moderate. The positive rate of diagnosis of LVDD based on the *Z* value criteria was higher than that based on the guidelines. Moreover, this study revealed that the consistency strength of the two sets of criteria in diagnosing LVDD in low-risk HF was poor, however, the overall difference was not statistically significant. Additionally, this showed that the consistency between the two sets of criteria for grading LVDD in children with reduced LVEF was moderate, and the severity of LVDD based on the *Z* value criteria was higher than that based on the guidelines.

Children are at a growth stage, and their heart size and function are also in development; therefore, the normal reference values of children's hearts cannot be the same as those of adults. This study showed that the normal reference value range of children based on BSA is more stable than age, sex, race, etc. (15). Regarding the construction of the normal reference range for children's Echocardiography, research has been gradually optimized over an extended period, leading to a consensus on a normal reference range based on the *Z* value (15–28). However, owing to the differences in research scales, age distributions and system construction methods, the *Z* value of the same measurement may vary in different systems (15). The reference range for the normal value in this study was derived from a study on the normal reference values of a children's echocardiographic measurement indexes based on 1,631 healthy children and 300 newborns, encompassing the largest data volume and the most comprehensive measurement indexes in China; performed by Shenzhen Children's Hospital with high reliability.

The elevation of left ventricular filling pressure indicated by cardiac catheterisation is the gold standard for the diagnosing LVDD. As early as 1997 (29, 30), some researchers have studied the correlation between the E peak of mitral valve flow, *E*/*A* and *E*/*e*′ and left ventricular filling pressure, suggesting that *E*/*e*′ has the strongest correlation with invasive left ventricular filling pressure parameters. Since then, many studies have explored the correlation between *E*/*e*′ and invasive left ventricular filling pressure parameters. Although there are differences in sensitivity and specificity among various studies, the value of *E*/*e*′ in diagnosing LVDD has been recognized by most researchers. Caballero et al. (31) reported that *E*/*e*′ is relatively stable and is little influenced by growth and development, suggesting that this indicator is very suitable for growing children. This study demonstrated that the consistency strength of the two criteria for mitral annular septal *E*/*e*′ evaluation was moderate, whether in the HF group or control group, which further confirmed the stability and reliability of *E*/*e*′ in the evaluation of LVDD.

Left atrial dilation is considered the result of a long-term increase in left atrial pressure. The increase in the left atrial maximum volume index may indicate LVDD. The correlation coefficients between the maximum volume index of the left atrium and invasive filling pressure parameters across studies (32, 33), and the efficiency of diagnosing the increase in left ventricular filling pressure is also inconsistent (34–36). This finding suggests that the left atrial maximum volume index is unstable for evaluating LVDD. This study demonstrated that the two criteria had poor consistency in evaluating the left atrial maximum volume index. The reasons for this are as follows: (1) The left atrial maximum volume index changed significantly with age. (2) The measurement variation between the observers was large. (3) The number of samples required to obtain a normal reference value was insufficient. As few studies on the normal reference value of children's left atrial maximum volume index exist, a multicentre study with large sample data is required for verification.

Tatiana Kuznetsova et al. (37) evaluated the LVDF in 1,407 community residents by using Echocardiography. Two evaluation criteria were used, the age-specific cut-off value based on the population and the cut-off value recommended by the 2016 ASE/EACVI guidelines. The positive rate and severity of LVDD based on the age-specific cut-off value were higher than those based on the guidelines, and the value that predicted adverse cardiovascular events was also higher for the age-specific value than that of the standard based on the guidelines. The present study revealed that the positive rate of diagnosis of LVDD in children with HF and a high risk of HF based on the BSA-transformed *Z* value standard was higher than that of the standard based on the guidelines. The severity of LVDD based on the BSA-transformed *Z* value standard was also higher than that of the standard based on the guidelines, similar to results of the research in adults. This study also found that in children with a low risk of HF, no case of LVDD was found based on either of the two evaluation criteria, thus indicating high specificity. This finding suggests that the BSA-transformed *Z* value standard may be more conducive to the early recognition of LVDD in children with HF and those with high risk of HF.

## Conclusion

The evaluation of LVDF in children with HF and in those with a high risk of HF based on the BSA-transformed *Z* value standard may be more helpful in the early identification of LVDD.

## Limitation

As most of the patients did not receive cardiac MRI or catheters examinations, the accuracy of the two criteria could not be compared.

## Data Availability

The raw data supporting the conclusions of this article will be made available by the authors, without undue reservation.

## Supplementary material

Supplementary Table 1

Cut-off values of ±*Z* values corresponding to each Echocardiography parameter. BSA, body surface area; E, Early peak velocity of mitral valve in diastole; *E*/*A*, ratio of early diastolic peak velocity of mitral valve to late diastolic peak velocity; *e*′, early peak diastolic velocity of mitral annulus; *E*/*e*′, ratio of early peak diastolic velocity of mitral annulus to early peak diastolic velocity of mitral annulus; SD, standard deviation
