# Supplementary material for: Comparison of two echocardiography-based methods for evaluating pediatric left ventricular diastolic dysfunction
Source: Front Pediatr. 2023 Sep 4;11:1206314. doi: 10.3389/fped.2023.1206314 (PMC10507386; doi:10.3389/fped.2023.1206314)
Supplement: Supplementary file 1 [file Table1.pdf]

Supplementary Table 1

		Left atrial maximum volume index			Mitral valve E peak			Mitral E/A				Mitral Annular Septal E/e'			Mitral Annulus Septal Wall e'			Mitral Annulus Lateral Wall e'		
		**mean (ml/m2)**	**SD**	**+2Z**	**mean (m/s)**	**SD**	**-2Z**	**mean**	**SD**	**-2Z**	**+2Z**	**mean**	**SD**	**+2Z**	**mean (cm/s)**	**SD**	**-2Z**	**mean (cm/s)**	**SD**	**-2Z**
**BSA(m^2)**	0.25-0.3	12.55	3.39	19	1.03	0.15	0.7	1.26	0.3	0.7	1.8	11.64	3.36	18	9.32	2.19	5	10.66	2.03	7
	0.31-0.4	13.47	2.64	19	1.09	0.13	0.8	1.42	0.25	0.9	1.9	10.12	1.83	14	11	1.71	8	11.7	1.92	8
	0.41-0.5	13.05	2.76	19	1.13	0.15	0.8	1.47	0.26	1	2	10.1	1.83	14	11.42	1.8	8	13.38	2.16	9
	0.51-0.6	12.44	2.87	18	1.17	0.14	0.9	1.63	0.33	1	2.3	9.25	2.05	13	12.9	1.74	9	15.3	2.42	10
	0.61-0.7	13.34	3.01	19	1.14	0.15	0.8	1.57	0.27	1	2.1	8.4	1.66	12	13.89	2.18	10	16.37	3	10
	0.71-0.8	13.17	2.67	19	1.16	0.17	0.8	1.62	0.36	0.9	2.3	8.62	1.56	12	13.62	2.09	9	16.27	3.33	10
	0.81-0.9	14.29	3.16	21	1.12	0.15	0.8	1.59	0.33	0.9	2.3	8.33	1.71	12	13.69	1.8	10	16.65	3.53	10
	0.91-1.0	13.42	3.32	20	1.05	0.15	0.8	1.69	0.39	0.9	2.5	7.93	1.74	11	13.59	2	10	16	3.4	9
	1.01-1.1	14.01	3.27	21	1.03	0.2	0.6	1.56	0.33	0.9	2.2	7.63	1.94	12	13.92	2.71	9	16.05	3.6	9
	1.11-1.2	13.64	2.79	19	1.02	0.14	0.7	1.62	0.38	0.9	2.4	7.51	1.61	11	13.98	2.51	9	17.17	3.67	10
	1.21-1.3	14.1	1.99	18	1.06	0.19	0.7	1.71	0.35	1	2.4	7.31	1.67	11	14.79	2.57	10	17.62	3.52	11
	1.31-1.4	13.52	2.6	19	1.1	0.21	0.7	1.61	0.39	0.8	2.4	7.58	2.11	12	14.97	2.37	10	17.39	2.9	12
**Newborn weight**																				
**(kg)**	1.5-2	8.01	2.34	13	0.61	0.15	0.3	1.14	0.22	0.7	1.6	13.18	2.67	19	5	1.41	2	5.84	2.32	1
	2.1-2.5	9.97	3.89	18	0.68	0.17	0.3	1.15	0.27	0.6	1.7	12.74	3.23	19	5.62	1.5	3	7.04	1.91	3
	2.6-3	9.16	3.1	15	0.7	0.15	0.4	1.2	0.27	0.7	1.7	12.42	4.17	21	5.9	1.91	2	6.95	2.35	2
	3.1-3.5	9.64	3.6	17	0.77	0.16	0.5	1.18	0.26	0.7	1.7	12.11	3.89	20	6.72	2.24	2	7.6	2.17	3
	3.6-4	10.93	3.72	18	0.85	0.19	0.5	1.15	0.24	0.7	1.6	13.32	3.78	21	6.7	2.12	2	8.05	2.62	3
	4.1-4.5	11.49	3.47	18	0.83	0.14	0.6	1.18	0.19	0.8	1.6	12	2.65	17	7.17	2.32	3	9.18	2.54	4
	4.6-5	11.34	2.94	17	0.91	0.18	0.6	1.18	0.26	0.7	1.7	13.58	2.51	19	7.07	1.14	5	9.36	1.55	6
